# Improving survival models in healthcare: a novel matching approach

**DOI:** 10.21203/rs.3.rs-5467577/v1

**Published:** 2024-12-12

**Authors:** Dimitris Bertsimas, Catherine Ning, Per Eystein Lønning, Hideo Baba, Itaru Endo, Richard Burkhart, Federico N Aucejo, Felix Balzer, Martin E Kreis, Georgios Antonios Margonis

**Affiliations:** 1 Operations Research Center, Massachusetts Institute of Technology, Cambridge, MA, USA.; 2 Department of Clinical Science, University of Bergen, Department of Oncology, Haukeland University Hospital, Bergen, Norway.; 3 Department of Gastroenterological Surgery, Graduate School of Medical Sciences, Kumamoto University, Kumamoto, Japan.; 4 Department of Gastroenterological Surgery, Yokohama City University Graduate School of Medicine, Yokohama, Japan.; 5 Department of Surgery, Johns Hopkins University School of Medicine, Baltimore, Maryland, USA.; 6 Department of General Surgery, Digestive Disease Institute, Cleveland Clinic, Cleveland, Ohio, USA.; 7 Charité - Universitätsmedizin Berlin, Berlin, Germany.; 8 Department of General and Visceral Surgery, Charité Campus Benjamin Franklin, Berlin, Germany.; 9 Department of Surgery, Memorial Sloan Kettering Cancer Center, New York, NY, USA.

## Abstract

We present, to our knowledge, the first methodological study aimed at enhancing the prognostic power of Cox regression models, widely used in survival analysis, through optimized data selection. Our approach employs a novel two-stage mechanism: by framing the prognostic stratum matching problem intuitively, we select prognostically representative patient observations to create a more balanced training set. This enables the model to assign equal attention to distinct prognostic subgroups. We demonstrate the methodology using an observational dataset of 1,799 patients with resected colorectal cancer liver metastases, 1,197 of whom received adjuvant chemotherapy and 602 who did not. In our study, as is current standard practice, the comparator was training prognostic models on the entire cohort (referred to as ”model 1”). Models trained on the untreated and treated subgroups, matched through our approach (referred to as ”model 3”), showed an improvement of up to 20% in bootstrapped C-indices compared to model 1. Notably, model 3 exhibited superior calibration, with a 6- to 10-fold improvement over model 1. Additional performance metrics aligned with these findings, and robustness was confirmed through bias-corrected bootstrapping. Given the ongoing development of numerous linear prognostic models and the general applicability of our approach to any observational data, this method holds significant potential to impact biomedical research and clinical practice where prognostic models are utilized.

## 1. Introduction

Prognostication, the act of foretelling future outcomes, is a key component in decision making both for patients and doctors. Accurate prognostication informs patients about their realistic odds of surviving and facilitates the discussion between clinicians and patients on the cost-effectiveness of a particular medical intervention, weighing its benefits against competing risks of toxicity, morbidity, and mortality. While linear prognostic models have long been used in clinical practice to predict time-to-event (TTE) outcomes, the advent of high-performance machine learning software and the growing availability of big data offer the potential for even more sophisticated prognostic models. In both traditional and modern approaches, the entire clinical dataset is typically used to train the model.

This common practice — training a single model on the entire cohort — works well for randomized controlled trials (RCTs), but when applied to observational data, it introduces confounding bias, undermining reliable outcome prediction. Specifically, patients in observational datasets often have varying propensities to receive certain treatments, leading to imbalances between treated and untreated groups in terms of prognostic variables. As a result, prognostic models may disproportionately focus on densely populated areas of the data, rather than giving balanced attention across the entire cohort.

To address this imbalance, we propose a novel two-stage approach. In the first stage, we apply a matching technique to create a balanced cohort of treated and untreated patients. In the second stage, we train separate prognostic models on these matched groups. While matching treated and untreated patients is a common technique for estimating average treatment effects in observational data, its application to prognostication has not been previously explored. We believe this gap exists due to a traditional lack of attention to confounding bias in prognostication studies, as well as the common perception that reducing the size of the training set necessarily weakens prognostic model performance.

We propose a novel two-stage mechanism that aims to ensure that the model gives equal attention to all prognostic subgroups. In light of this, we formulate our main hypotheses as follows:

1. Our matching algorithm, applied to patient subgroups stratified by prognostic scores, allows to match on not only observed patient characteristics but also latent confounding factors. This approach goes beyond conventional matching techniques such as propensity score matching (PSM).
2. By retaining only a carefully selected subset of patients, our matching algorithm creates a balanced cohort, with equal numbers of treated and untreated subjects in each prognostic stratum (e.g., high-, medium-, low-risk). This enables the model to focus equally across different prognostic areas. Unlike traditional approaches, where a single model is trained on the entire cohort, we demonstrate how selective data reduction can enhance prognostic accuracy.

We test our hypotheses using a large, multi-institutional dataset of patients with colorectal cancer liver metastases (CRLM) treated at tertiary academic centers, some of whom received adjuvant chemotherapy. To validate our modeling approach, rather than a specific model, we assess both discrimination and calibration metrics using the bias-corrected bootstrap technique, as proposed by Frank Harrell. A recent systematic review of 71 prognostic models for CRLM (Kokkinakis et al., 2024) found that all models were trained on the entire cohort and none employed propensity matching or any other form of matching).[1] Thus, the primary comparator to our approach is training models on the entire cohort,

## 2. Methods and Materials

### 2.1 Prognostic Models

**Figure 1:**
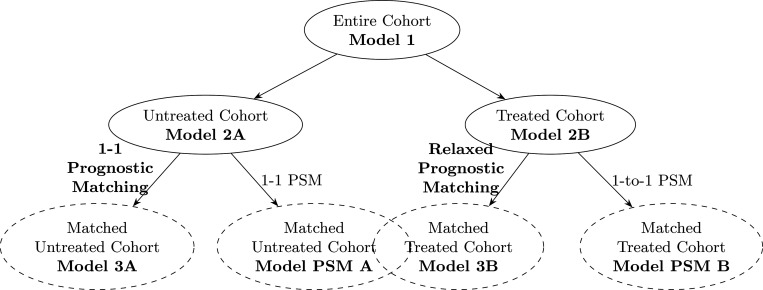
Visual abstract of the prognostic Cox regression models trained on different cohorts and compared against each other for discrimination and calibration power.

The most abundantly used model for survival analysis in biomedical literature, including CRLM, is the Cox Proportional Hazard (CPH) linear regression model [1, 2] All the models developed in this work are CPH models with five-year overall survival (OS) as endpoint. The five-year endpoint was chosen because it is a standard timeframe in oncological studies. We start by presenting our nomenclature to distinguish our proposed methodology from traditional approaches, followed by a visual summary in Fig.1:

- **Model 1** is the Cox model trained on the entire available cohort, which is the standard and most wide-spread approach in the CRLM prognostication literature [1]. For fair comparison against the other models, we include the treatment as a binary training variable despite that very few models of this type do so in practice.
- **Model 2A** is the Cox model trained on the untreated sub-cohort only and **Model 2B** the Cox model trained on the treated sub-cohort. While training models separately for treatment groups is uncommon for medical prognostication, this more granular approach may enhance predictive accuracy, as patients receiving different treatments often exhibit distinct characteristics that can affect outcomes differently.
- **Model 3A** is the Cox model trained on the matched untreated sub-cohort only and **Model 3B** the Cox model trained on the matched treated sub-cohort, which are subsets of the training sets for Models 2A and 2B as we have discarded data via prognostic stratum matching. This is our proposed 2-stage mechanism in which we first construct the strata from the baseline probabilities of death within five years and perform matching between the treated and untreated patients. In the second stage, we train separately two Cox models on the matched untreated and matched treated sub-cohorts.
  For comparison against conventional matching techniques, we also create a training set after conducting 1:1 nearest neighbor propensity score matching (PSM) without replacement with a propensity score estimated using first a random forest model of the treatment on the covariates, then as an additional benchmark, also a generalized linear model [3]. We fit **Model PSM A** and **Model PSM B** separately on these matched untreated and treated data, respectively, and assess them in the same way as the other models.

### 2.2 Matching

The goal of our proposed prognostic stratum matching approach is two-fold: firstly, it should select a balanced set of “prognostically representative” patients who not only share similar baseline risks of death within five years but also similar patient characteristics; secondly, the number of patients in each stratum after matching should be approximately equal to allow Models 3A and 3B to focus equally on different prognostic areas. Depending on the data at hand, the proportion of discarded data out of the original data can be large. To remedy the potential loss of modeling power caused by a too small training size, we may reinforce the 2-stage mechanism with an oversampling step after the matching process, where we duplicate or triple the matched data. Intuitively, data oversampling preserves the benefits of the matching (model focuses equally on a all prognosis areas) while compensating for the loss of modeling power through artificially increasing the number of training samples. Algorithm 1 summarizes the first stage of our methodology to create the training sets from prognostic stratum matching^1^.

**Algorithm 1:** Stratification and Matching

1: **Input:** Training set D (with covariates X, binary treatment vector a, time to event vector t, and event vector Y)
2: **Output:** Matched treated and untreated subsets (without oversampling step)
3: Partition the training set D into untreated subset $D_{a=0}$ and treated subset $D_{a=1}$.
4: Fit a CPH on $D_{a=0}$, apply it on D to estimate the baseline risk of death within 5 years for the entire cohort, P(Y=1∣X,t≤5).
5: Stratify $D_{a=0}$ and $D_{a=1}$ by risk deciles into 10 prognostic strata $D_{i\in 1:10,a\in \{0,1\}}$.
6: Find $\alpha \leftarrow \text{min}\{\text{nrow}\left(D_{i,a=0}\right),\;\text{nrow}\left(D_{i,a=1}\right),\;\text{∀}i\in 1:10\}$ s.t. α≥20 (else find the next smallest α)
7: **for** i=1 : 10 **do**
8: $m\leftarrow \{\text{nrow}\left(D_{i,a=0}\right),\;\text{nrow}\left(D_{i,a=1}\right)\}$, $n\leftarrow \text{max}\{\text{nrow}\left(D_{i,a=0}\right),\;\text{nrow}\left(D_{i,a=1}\right)\}$.
9: $A\leftarrow \text{min}\{D_{i,a=0},D_{i,a=1}\}$, $B\leftarrow \text{max}\{D_{i,a=0},\;D_{i,a=1}\}$.
10: **for** each i∈A and j∈B **do**
11: $d_{ij}$ ← root-sum-square of the differences between normalized covariates $\hat{X}$
12: **end for**
13: **if** m≤α **then**
14: $\left(D_{i,a=0}^{matched},\;D_{i,a=1}^{matched}\right)$ ← solve basic 1-to-1 prognostic matching problem 2.
15: **else**
16: $D_{i,a=0}^{matched}$ ← solve 1–1 equalized prognostic matching problem 1
17: $D_{i,a=1}^{matched}$ ← relaxed prognostic matching problem 3.
18: **end if**
19: **end for**
20: **return** $\left(D_{a=0}^{matched},\;D_{a=1}^{matched}\right)$

In the following, we introduce three versions of the optimization problem for prognostic stratum matching in the last steps of Algorithm 1. Common to all of them is the choice of binary decision variables $z_{ij}$ to encode which patients to retain or discard: $z_{ij}=1$ if patient i is matched to patient j, otherwise $z_{ij}=0$. The objective function is to minimize the total distance across selected pairs of patients, or in other words maximize similarity of the matched patient characteristics. Thus, we take the Euclidean distance between the normalized feature vectors of the untreated patients and those of the treated patients in a given risk stratum. Within a stratum, we label the treatment group with fewer patients as A and the group with more patients as B. Finally we denote by α the minimum size for a stratum to be sufficiently large for data reduction. This is chosen as the size of the smallest or next-to-smallest stratum subgroup (treated or untreated), provided it is above some reasonable cut-off value (e.g. 20).

#### 2.2.1 1–1 Equalized Prognostic Stratum Matching

In the 1–1 prognostic matching problem, each patient in a given risk stratum $D_{i}$ from one treatment group is matched to at most one patient from the other treatment group in the same stratum. There are two versions of formulating the 1–1 matching problem in this section. The first version mainly serves the purpose of discarding patients from either treatment group until the same number of treated and untreated patients is reached across different strata, a process we call *equalization*. This requires $D_{i}$ to contain at least α patients in A before matching (m≥α in Algorithm 1). Since the optimization program will be minimizing the number of selected pairs, when we upper bound the number of matches per patient by 1 (Constraints 1 and 2), we need to lower bound the number of matched pairs in a stratum by some threshold value. By setting the latter to be α for all strata, Constraint 3 enforces that the total number of matched patients is the same across the different risk strata (hence these are *equalized*), achieving our desired goal of equal representation from all the prognostic areas. The above specifications lead to the following problem formulation:

(Objective) $$\underset{z}{\text{min}}\sum_{i\in A}\sum_{j\in B}d_{ij}z_{ij}$$

(Constraint 1) $$\text{s.t.}\;\sum_{j\in B}z_{ij}\leq 1\;\text{∀}i\in A$$

(Constraint 2) $$\sum_{i\in A}z_{ij}\leq 1\;\text{∀}j\in B$$

(Constraint 3) $$\sum_{i\in A}\sum_{j\in B}z_{ij}\geq \alpha$$

(Distance) $$d_{ij}=\sqrt{\sum_{k=1}^{K}{\left({\overset{ˆ}{x}}_{ik}-{\overset{ˆ}{x}}_{jk}\right)}^{2}}$$

(Normalization) $${\overset{ˆ}{x}}_{ik}=\frac{x_{ik}-{\overset{‾}{x}}_{k}}{\sigma_{k}}$$

(1) $$z_{ij}\in \{0,1\}$$

where ${\overset{‾}{x}}_{k}$ and $\sigma_{k}$ are the mean and standard deviation of the feature vector $x_{k}$ of the patients in either group A or B.

On the other hand, if m≤α, we cannot afford to discard too much data if we want to ensure proper representation of that prognostic area. We modify Constraint 1 such that each patient in the minority group A is paired up with exactly one patient from B, i.e. we may discard data from group B but not from A until we reach the same amount of patients in both groups (hence Constraint 3 is no longer needed). The second version of 1–1 matching, designed for prognostic strata with an insufficient number of observations, is thus formulated as follows:

(Objective) $$\underset{z}{\text{min}}\sum_{i\in A}\sum_{j\in B}d_{ij}z_{ij}$$

(Constraint 1) $$\text{s.t.}\;\sum_{j\in B}z_{ij}=1\;\text{∀}i\in A$$

(Constraint 2) $$\sum_{i\in A}z_{ij}\leq 1\;\text{∀}j\in B$$

(Distance) $$d_{ij}=\sqrt{\sum_{k=1}^{K}{\left({\overset{ˆ}{x}}_{ik}-{\overset{ˆ}{x}}_{jk}\right)}^{2}}$$

(Normalization) $${\overset{ˆ}{x}}_{ik}=\frac{x_{ik}-{\overset{‾}{x}}_{k}}{\sigma_{k}}$$

(2) $$z_{ij}\in \{0,1\}$$

#### 2.2.2 Relaxed Prognostic Stratum Matching

We further propose a relaxed version of our problem formulation where we relax the upper bounds in Constraints 1 and 2 such that one patient can be matched to up to two patients from the other group. Constraint 3 still enforces that the total number of selected pairs in different risk strata is the same, however the selection may now be performed with replacement:

(Objective) $$\underset{z}{\text{min}}\sum_{i\in A}\sum_{j\in B}d_{ij}z_{ij}$$

(Constraint 1) $$\text{s.t.}\;\sum_{j\in B}z_{ij}\leq 2\;\text{∀}i\in A$$

(Constraint 2) $$\sum_{i\in A}z_{ij}\leq 2\;\text{∀}j\in B$$

(Constraint 3) $$\sum_{i\in A}\sum_{j\in B}z_{ij}\geq \alpha$$

(Distance) $$d_{ij}=\sqrt{\sum_{k=1}^{K}{\left({\overset{ˆ}{x}}_{ik}-{\overset{ˆ}{x}}_{jk}\right)}^{2}}$$

(Normalization) $${\overset{ˆ}{x}}_{ik}=\frac{x_{ik}-{\overset{‾}{x}}_{k}}{\sigma_{k}}$$

(3) $$z_{ij}\in \{0,1\}$$

#### 2.2.3 Comparison to 1–1 Propensity Score Matching

Propensity score matching is the most frequently used algorithm for matching in biomedical studies and selects nearest neighbour samples of the original treated and untreated groups with similar covariate distributions. The similarity metric is based on estimated propensity scores for receiving the treatment (here, the adjuvant chemotherapy variable) using binary classifiers such as random forest (RF) or generalized linear models (GLM) in our implementation. Treated and untreated patients are matched in the standard 1–1 manner for the same covariates used to match in the prognostic stratum matching, without equalizing across strata. Thus the untreated cohort, which before matching contains less patients in the first place, remains the same after matching. On the other hand, the PSM model using GLM is parameterized such that it discards patients from both untreated and treated cohorts.

### 2.3 Validation and Performance Metrics

The conventional method for model validation in machine learning, in the absence of independent external test data, is to hold out a fraction from the original dataset as test set, also known as data splitting. In chapter 5 of Frank Harrell’s textbook on regression modelling strategies [4], data splitting is deplored for being an unstable method for validating models due to the randomness underlying the train-test split, especially when the number of subjects falls short of 20000. Another problem arises when feature selection is performed prior to model fitting as data splitting validates *only one example of many potential models*.

Instead, resampling is a more reliable technique when the goal is to validate *not the model* per se, but *the process* that was used to develop the model. Resampling does not set aside a valuable portion of the original dataset for out-of-sample testing, and the randomness becomes negligible due to the large number of resamples. Moreover, it is honest in reporting the results because it depicts the uncertainty in feature selection, e.g., the disagreements in which variables are selected from one resample to the next. For our experiments on a relatively small dataset of CRLM patients, the bootstrap technique allows us to assess the effect of our prognostic stratum matching process on the model performance in a non-arbitrary manner. Since ordinary bootstrapping may yield overly optimistic estimates (e.g., when the ratio of number of observations to the number of selected covariates is small), we implement a bias-corrected variation introduced by Efron and outlined in Section 5.3.5 of [4]. It subtracts from the original performance index an estimate of the expected value of the optimism, which thus provides an estimate of the expected bias-corrected index.

Next, we list a number of industry-standard as well as relatively state-of-the-art performance metrics used in our empirical analyses. In survival analysis, we distinguish between discrimination and calibration metrics:

- Discrimination metrics measure the ability of the model to correctly differentiate between subjects who experience the event (for our example, death within five years) and those who do not. They can be a variant of the expected squared error and/or express the correlation between predicted and actual outcomes. Concordance indices, also known as C-statistics, are typically applicable for censored time-to-event binary diagnostic outcomes and provide a global assessment of the fitted survival model.
- In contrast, calibration metrics focus on the prediction of the t-year survival for a fixed time. We inspect mainly two types of calibration [5]:
  - calibration-in-the-large, which quantifies the ratio of the number of observed events in the validation sample to the number of events predicted by the regression model,
  - moderate calibration, which quantifies the extent to which the observed event rate equals the predicted risk among patients with the same predicted risk.

Because a model with high discriminatory power can still exhibit poor calibration, we will consider a mix of both types of metrics to assess the effectiveness of our proposed methodology on model training in a comprehensive way, namely:

1. **Harrell’s C index** [6] represents the estimated conditional probability that for any pair of “event” and “no event”, the predicted risk of the event is higher for the former (such a pair is *concordant*). Censored observations are included in the calculation of the index and can be adjusted for using a Kaplan-Meier estimator, but this may also introduce bias if the censoring is heavy.
2. **Uno’s C index** [7] is a more novel concordance measure and, like the Harrell’s C index, checks for concordance based on the ranks of the predicted risks at each event time. However, it also attempts to correct for the bias due to the study-specific censoring distribution through the technique of inverse probability of censoring weights (IPCW). In pairs where at least one subject is censored, the IPCW weights are applied, which are the inverse of the probability of being uncensored for the subject at each time point estimated using a Kaplan-Meier or similar estimator. Effectively, observations with a higher probability of being censored receive more weight, which may compensate for the fact that they are underrepresented in the data.
3. **Brier score** [8] measures the mean squared error between the predicted survival probabilities and the actual survival outcomes at a specific time point. It is not a measure of either discrimination performance or calibration performance alone, but a measure of overall performance.
4. The **calibration slope**, typically illustrated in calibration plots, quantifies the difference between the calibration curve and the diagonal line of best fit, hence a better calibrated model has a slope closer to unity.
5. The **Integrated Calibration Index** (**ICI**) [9] calculates the mean absolute difference between observed and predicted survival probabilities, the $E_{50}$ is the median, the $E_{50}$ the 90*^th^* percentile, and $E_{max}$ the maximum absolute difference. The greatest utility of these rather novel metrics is their ability to provide comparison of relative calibration between different prediction models. The ICI is effectively the *weighted* absolute difference between the calibration curve and the diagonal line of best fit where the weights correspond to the predicted probabilities.
6. The **Observation-Estimation** [10] (OE) score is the ratio between mean observed and mean predicted risk.

### 2.4 Statistical Analysis and Software Specifications

We have selected our prognostic variables for Cox analysis based on clinical knowledge and existing literature and not by step-wise selection methods to avoid instability and over-estimations [11]. Categorical variables are reported as counts and percentages while continuous variables are presented as medians with interquartile ranges (IQRs). Categorical and continuous variables are compared with the $\chi^{2}$ test and the Mann-Whitney U test, respectively. We use Iterative Imputer for continuous and Simple Imputer for categorical variables from the scikit-Learn library to perform multiple imputations under the missing at random assumption (Python v3.11.5).

The Kaplan-Meier method and log-rank test are used for uni-variable survival analysis, including the calculations of follow-up quantiles and overall survival times. All Cox models and Kaplan–Meier estimators are implemented from the rms, survminer, and survival packages in the R 4.3.2 programming language. All prognostic stratum matching problems are solved with the Gurobi 11.0.0 optimizer^2^ in the Julia 1.9.4 programming language to obtain the matched cohorts for Models 3A and 3B. For 1:1 nearest neighbor propensity score matching, the MatchIt library in R was used to obtain the matched cohorts for PSM A and PSM B. The matching was first based on distances estimated by a random forest model, and then a generalized linear model (GLM) was trained to estimate the distance measure, with the caliper set to 0.2.

The Harrell’s C index and calibration slope are readily obtained from the bootstrap validation function in the rms package^3,4^ whereas the rest are computed from manual bootstrapping, both approaches using *N* = 100 resamples.

All computing was performed on the personal MacBook Apple M2 Pro 16GB Memory.

## 3 Results

### 3.1 Data Specifications

**Figure 2:**
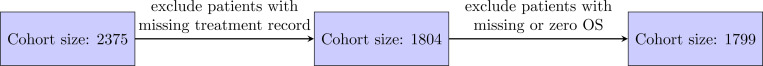
Flowchart of inclusion criteria for study cohort.

**Figure 3:**
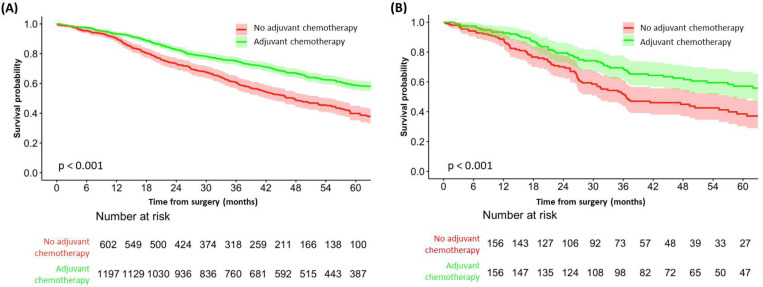
Kaplan-Meier plots (A) before matching and (B) after 1–1 equalized prognostic stratum matching

In this observational cohort study, we considered 2375 adult patients who underwent surgery for colorectal cancer liver metastases. Patients were included if they had complete records for adjuvant chemotherapy status and overall survival time, as these variables were deemed inappropriate for imputation if missing. This resulted in a final cohort of 1,799 eligible patients, as shown in the flowchart in Figure 2. Supplemental Table S1 and Table 1 present the baseline characteristics, comparing treated and untreated CRLM patients before and after imputations, respectively. The median OS was 66.5 months, with 1-year, 3-year, and 5-year survival rates of 92.33%, 70.20%, and 52.59%, respectively. The median follow-up time was 61.4 months (IQR: 35.6–96 months). Kaplan-Meier survival curves in Figure 3 illustrate the survival rates before and after prognostic stratum matching.

Based on a thorough review of the relevant literature, we selected 10 patient characteristics as predictor variables for training all Cox models [12]. These variables include age, diameter of the largest CRLM tumor, number of tumors, carcinoembryonic antigen (CEA) level, T category of the primary tumor, primary lymph node involvement, primary tumor side, presence of extrahepatic disease, surgical margin status, and KRAS mutational status. We excluded primary tumor grade due to the large proportion of missing data for this variable.

### 3.2 Prognostic Stratum Matching

In the first stage of our 2-stage mechanism, we classify patients into 10 prognostic strata based on deciles of 5-year mortality risk. While finer stratification can potentially improve prediction accuracy, the primary constraint in practice is often the size of the training cohort. For this reason, we chose a granularity of 10 strata, striking a balance between maximizing predictive accuracy and maintaining a reasonable number of patients per stratum. As shown in Table 2 prognostic matching effectively corrected the statistically significant imbalances in nine of the thirteen prognostic variables between the treated and untreated groups (Table 1).

**Figure 4:**
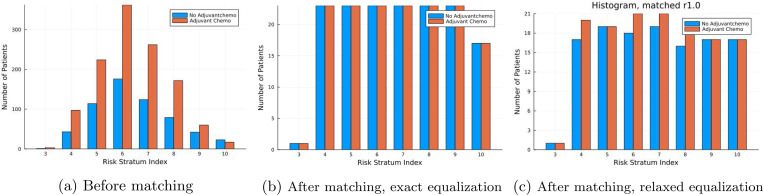
Prognostic stratum histograms of training patients before and after matching. The stratification is done by deciles hence a Risk Stratum Index of 3 corresponds to the probability of death range 0.2 − 0.3, an Index of 10 to 0.9 − 1.0 etc.

The histograms in Figure 4 illustrate the impact of our prognostic stratum matching process. First, it is important to note that the Cox model used for stratification (step 4 in Algorithm 1) predicts a predominantly medium risk of 5-year mortality for both untreated and treated cohorts. In this example, it becomes clear that we cannot set the threshold value α to the globally smallest stratum size, as stratum 3 contains only a single untreated patient. Instead we let α as the smallest stratum size greater than 20, which, in this case, is 23 from the untreated group in stratum 10. Given this α, we apply 1–1 matching without equalization (Problem 2, see 2.2.1–2.2.2) in strata 3 and 10 since *m* = 1 and *m* = 17 (the treated group in stratum 10) respectively. For the remaining strata, where m≥α, we implement both 1–1 equalized matching (1) and the relaxed prognostic stratum matching (3). The former results in a histogram showing that the number of patients is exactly equal across the different prognostic strata, while the latter produces a histogram with approximately equal numbers. Additionally, the histograms highlight how matching reduces the maximum disparity in the number of patients across strata. For example, the treated-to-untreated ratio in stratum 6 compared to stratum 3 is 350 : 2 before matching. After 1–1 equalized matching, the ratio is reduced to approximately 23 : 1. This provides an empirical intuition of how our prognostic stratum matching in the first stage enables the Cox model in the second stage to focus equally on low-, medium-, and high-risk prognosis areas during training.

### 3.3 Numerical Results

The sizes of the training cohorts for each Cox model are listed in Supplemental Table S2. Tables 3 and 4 present the in-sample performance metrics, evaluated on each model’s respective training set. Tables 5 and 6 display the bias-corrected performance metrics, evaluated using the bootstrapping validation method described in 2.3. When discussing our comparisons, we primarily assess the performance within the A models and B models separately before comparing both groups against Model 1. It is important to note that, for the PSM model using random forest (RF), no data from the untreated cohort is discarded during 1–1 propensity score matching, as this group constitutes the minority. Consequently, the performance of PSM A RF mirrors that of Model 2A. However, this does not apply to the results of the PSM model using GLM, which discards patients from both untreated and treated cohorts.

Our empirical results highlight the following:

- Models 3A and 3B which use our prognostic stratum matching approach had the highest in-sample and bootstrap discrimination metrics as shown in Tables 3 and 5. These models outperformed those trained on unmatched cohorts as well as those employing propensity score matching by a significant margin.
- When comparing the two versions of prognostic stratum matching, the relaxed matching approach showed noticeably higher discriminatory capacity than the 1–1 equalized matching when used in the treated patients. The opposite was noted in the untreated patients where 1–1 equalized matching approach showed higher discriminatory capacity, albeit the difference was less pronounced.
- In terms of calibration performance, model 3A which uses our 1–1 equalized prognostic stratum matching approach in the untreated patients had the highest bootstrap calibration metrics as shown in Table 6, with the exception of calibration slope. Model 3B which uses our relaxed matching approach in the treated patients performed worse than its competitors.
- We attribute the inferior calibration performance of Model 3B to the small sample size of its training cohort. To improve calibration, we applied our methodology with an additional oversampling step. By replicating the matched cohorts two or three times, we observed improvements in both the calibration and discrimination performance of Models 3A and 3B, regardless of the matching approach. For instance, tripling the training cohort size of Models 3A and 3B resulted in excellent calibration and discrimination performance, surpassing all competitor models, as shown in Table 6.

## 4 Discussion

Prognostication plays a central role in biomedical research, with numerous prognostic models already being employed in routine clinical practice to inform patient outcomes and guide clinical decision-making [13]. As such, improving these models has become a significant focus across medical disciplines. Traditionally, progress has been made by identifying new prognostic factors and incorporating them into linear models. However, this is often a slow process. For example, in colorectal cancer liver metastases, only one new routinely available prognostic factor has been identified over the past 15 years (KRAS mutational status)[14].

Another avenue for improvement lies in the development of more sophisticated models, including those based on machine learning (ML). Unfortunately, while these models offer potential, they often come with trade-offs. Black-box ML methods may improve discriminatory power but sacrifice interpretability [15]. Alternatively, interpretable models, such as decision trees, tend to show only modest improvements in discrimination over linear models. Moreover, ML models often suffer from poor calibration, a shortcoming that has been described as their “Achilles’ heel” [16].

Our approach diverges from these traditional paths. Rather than focusing solely on the choice of model or the incorporation of new covariates, we prioritize optimizing the data used to train these models. We hypothesize that prognostic performance could be enhanced not by using all available patient data but by selecting a numerically balanced set of *prognostically representative* patients. This requires having a sufficient pool of observations in each treatment group, providing many ”matching options” that allow us to pair only those who are well-matched. A numerically balanced set of representative patients would allow the model to focus equally on distinct prognostic subgroups. While removing a large portion of the data might intuitively seem to reduce statistical power, as evidenced by the suboptimal performance of propensity score matching, our results suggest that removing “noise” actually strengthens the “signal” in the data. Oversampling these *representative* patients further mitigates concerns about statistical power, with incremental oversampling improving the bootstrapped metrics of the models.

Indeed, although the matched cohorts comprised just one-sixth of the entire patient cohort, the models fitted in the matched cohorts demonstrated an improvement in bootstrapped C indices of up to 20% compared to models trained on the entire cohort. To put this into perspective, achieving a 20% improvement in C index for CRLM prognostic models is remarkable. The most popular current prognostic model for CRLM, which added three new variables (KRAS, TBS, and extrahepatic disease) and redefined another one (CEA levels), achieved only a 7% improvement in C index over its predecessor [17, 18, 19]. In contrast, our 20% improvement was achieved using the same covariates across all models.

Importantly, the models trained using our 2-stage mechanism also exhibit excellent calibration, with a 6 to 10-fold improvement over models trained using traditional methods. Calibration is often under-reported in prognostic model development [20]. In CRLM, for example, only about half (56%) of models report calibration, which highlights a broader deficiency in the biomedical literature [1]. It is a common misconception that a high C index or AUC necessarily indicates an accurate prognostic model. While excellent discrimination suggests that a model can rank patients by risk, the predicted probabilities may still be miscalibrated. In clinical practice, it is the actual predicted probability, not just the rank, that influences critical decisions—such as whether to offer adjuvant chemotherapy following CRLM resection.

The superior performance of our method compared to conventional techniques can—at least theoretically—be attributed to both the reduction of observed confounding and the numerically balanced distribution of patients across prognostic subgroups. As expected with real-world observational data, confounding bias was present in our study cohort, where patients with more favorable characteristics were more likely to receive adjuvant chemotherapy. This was evidenced by statistically significant imbalances in nine of the thirteen prognostic variables between the treated and untreated groups (Table 1). Prognostic matching effectively corrected these imbalances (Table 2). Notably, although statistically significant, the imbalances were relatively mild, as shown in plot 4a, where the treated-to-untreated ratio remained fairly constant across most prognostic strata, except for the highest-risk stratum, where treated patients were the minority. Therefore, in this demonstration, we attribute our method’s superior performance primarily to its ability to allow the prognostic model to focus equally on different prognostic areas by equalizing patient numbers across prognostic strata. An additional argument supporting this is that the models trained on propensity score-matched cohorts—designed to correct imbalances across covariates—failed to outperform the models trained on the entire cohort. Regarding our approach, we anticipate even greater improvements in clinical settings with more pronounced confounding bias. However, it is important to note that this approach addresses only observed confounding; unobserved confounding remains outside its scope, as is true for all existing methodologies.

On a technical level, this study has several strengths. First, we use two discrimination metrics, which is particularly appropriate when right-censoring is pronounced. Secondly, we employ newly introduced numeric calibration metrics (ICI, E50, and E90), which provide a more precise evaluation of model calibration compared to the traditionally used calibration slope. These new metrics are particularly valuable as they facilitate the comparison of calibration across different models for survival data. In contrast, most CRLM prognostic models rely on calibration curves, which only allow for visual inspection of agreement between predicted and observed outcomes. Among studies reporting on new CRLM prognostic models, only 15% employed more granular calibration metrics, and those that did primarily used the outdated Hosmer-Lemeshow (HL) goodness-of-fit test (10 studies, 14.1%) [1]. A small minority reported calibration slope (4 studies, 5.6%), and none tested calibration using the ICI, E50, or E90 metrics.

Additionally, we use bootstrapping to validate the robustness of our findings, a more appropriate method than the commonly used random train-test split [21]. Bootstrapping offers greater accuracy, especially when compared to data splitting and cross-validation techniques that do not involve a large number of repetitions [22]. We choose internal validation through bootstrapping instead of external validation due to the nature of our models. Specifically, rather than pre-specifying the models or perform variable selection, we decide to include all 10 variables across all models and approaches. External validation would have been more suitable if our goal had been to validate a specific pre-specified model. In our case, however, the focus is on validating the model development process rather than the model itself.

In conclusion, we propose that a “less is more” perspective to prognostication, focusing on the most representative patients and reducing noise, can enhance model performance. Since the proposed methodology is not case-specific, it has the potential to be applied to any observational data, making it suitable for a wide range of clinical settings. To our knowledge, this is the first approach aimed at improving prognostication by optimizing data pre-processing. Future studies that adopt this approach will help validate whether it delivers on the promise demonstrated in this study. If successful, this could have a profound impact on biomedical research, where a vast number of prognostic models are continually being developed.

## Supplementary Material

Supplement 1

## Figures and Tables

**Table 1:** Baseline characteristics of treated or untreated CRLM patients before prognostic stratum matching with imputation. DFI and tumor grade are not predictor variables and thus not imputed.

Characteristic	Treated (n (%))	Untreated (n (%))	P-value

**Total Patients**	1197	602	
**Age in years (IQR)**	60 (52.0–67.0)	65.0 (58.0–73.0)	< 0.001
**Sex**			
Male	713 (59.6%)	371 (61.6%)	
Female	484 (40.4%)	231 (38.4%)	0.39899
**Median CEA in μg/L (IQR)**	8.0 (3.0–40.0)	9.9 (3.4–44.6)	0.39502
**Median Diameter of Largest CRLM in cm (IQR)**	3.0 (2.0–4.0)	3.3 (2.0–4.0)	0.7041
**Median Number of CRLMs (IQR)**	2.0 (1.0–3.0)	2.0 (1.0–3.0)	0.2190

**T Category of Primary Tumor**			
0.0	1 (0.1%)	4 (0.7%)	
1.0	53 (4.4%)	8 (1.3%)	
2.0	203 (17.0%)	58 (9.6%)	
3.0	672 (56.1%)	387 (64.3%)	
4.0	268 (22.4%)	145 (24.1%)	< 0.001

**Primary Lymph Node Involvement**			
No Metastases	495 (41.4%)	210 (34.9%)	
Metastases	694 (58.0%)	381 (63.3%)	0.008

**Primary Tumor Side**			
Right	563 (47.0%)	173 (28.7%)	
Left	331 (27.7%)	238 (39.5%)	
Rectal	303 (25.3%)	191 (31.7%)	< 0.001

**Extrahepatic Disease**			
0.0	1078 (90.1%)	516 (85.7%)	0.0062
1.0	119 (9.9%)	86 (14.3%)	

**Surgical Margin Status**			
R0	1068 (89.2%)	458 (76.1%)	
R1	129 (10.8%)	144 (23.9%)	< 0.001

**KRAS Mutation**			
0.0	676 (56.5%)	398 (66.1%)	
1.0	521 (43.5%)	204 (33.9%)	< 0.001

**Table 2:** Baseline characteristics of treated or untreated CRLM patients after prognostic stratum matching.

Characteristic	Treated (n (%))	Untreated (n (%))	P-value

**Total Patients**	156	156	
**Age in years (IQR)**	62.0 (55.8–69.0)	66.0 (58.8–73.2)	< 0.001
**Median CEA in μg/L (IQR)**	7.2 (3.4–19.0)	8.0 (3.0–24.3)	0.9855
**Median Diameter of Largest CRLM in cm (IQR)**	3.0 (2.0–4.0)	3.0 (2.1–3.5)	0.0875
**Median Number of CRLMs (IQR)**	1.0 (1.0–2.4)	1.0 (1.0–2.0)	0.9450

**T Category of Primary Tumor**			
0	0 (0.0%)	1 (0.6%)	
1	2 (1.3%)	1 (0.6%)	
2	17 (10.9%)	12 (7.7%)	
3	107 (68.6%)	116 (74.4%)	
4	30 (19.2%)	26 (16.7%)	0.5842

**Primary Lymph Node Involvement**			
No Metastases	47 (30.1%)	49 (31.4%)	
Metastases	109 (69.9%)	107 (68.6%)	0.8062

**Primary Tumor Side**			
Right	66 (42.3%)	67 (42.9%)	
Left	51 (32.7%)	50 (32.1%)	
Rectal	39 (25.0%)	39 (25.0%)	0.9913

**Extrahepatic Disease**			
0	121 (77.6%)	122 (78.2%)	
1	35 (22.4%)	34 (21.8%)	0.8915

**Surgical Margin Status**			
R0	135 (86.5%)	129 (82.7%)	
R1	21 (13.5%)	27 (17.3%)	0.3465

**KRAS Mutation**			
0	81 (51.9%)	77 (49.4%)	
1	75 (48.1%)	79 (50.6%)	0.6506

**Table 3:** In-sample discrimination metrics with best metrics among A or B models in bold.

Model	Harrell C	Uno C	Brier Score

1	0.6144 [0.5931, 0.6356]	0.5932 [0.5627, 0.6238]	0.2258
1A	0.6142 [0.5795, 0.6489]	0.6007 [0.5662, 0.6353]	0.2236
2A	0.6264 [0.5918, 0.6609]	0.6085 [0.5728, 0.6443]	0.2200
PSM A RF	0.6264 [0.5918, 0.6609]	0.6085 [0.5728, 0.6443]	0.2200
PSM A GLM	0.6222 [0.5872, 0.6573]	0.6065 [0.5700, 0.6430]	0.2210
3A 1–1	**0.6725** [**0.6125, 0.7325**]	**0.6780** [**0.6216, 0.7344**]	**0.1867**
3A relaxed	0.6619 [0.5974, 0.7263]	0.6662 [0.6085, 0.7240]	0.1910
1B	0.5795 [0.5523, 0.6067]	0.5566 [0.5148, 0.5984]	0.2319
2B	0.5806 [0.5531, 0.6080]	0.5596 [0.5189, 0.6004]	0.2296
PSM B RF	0.5881 [0.5501, 0.6262]	0.5847 [0.5475, 0.6220]	0.2306
PSM B GLM	0.5912 [0.5513, 0.6310]	0.5696 [0.5312, 0.6080]	0.2371
3B 1–1	0.6519 [0.5794, 0.7245]	0.6377 [0.5676, 0.7079]	0.2079
3B relaxed	**0.6993** [**0.6298, 0.7688**]	**0.6815** [**0.6029, 0.7602**]	**0.2033**

**Table 4:** In-sample calibration metrics with best metrics among A or B models in bold

Model	OE	ICI	E50	E90	Emax	Calibration Slope

1	**0.9869** [0.9150, 1.0645]	0.0049	0.0049	0.0086	0.0187	1.0000 [**0.8401, 1.1599**]
2A	**1.0013** [0.8904, 1.1260]	0.0152	0.0138	0.0310	0.0607	1.0000 [0.7601, 1.2399]
PSM A RF	1.0013 [0.8904, 1.1260]	0.0152	0.0138	0.0310	0.0607	1.0000 [0.7601, 1.2399]
PSM A GLM	1.0034 [0.8902, 1.1311]	0.0148	0.0137	0.0305	0.0617	1.0000 [0.7464, 1.2536]
3A 1–1	1.0233 [0.8197, 1.2776]	0.0087	0.0082	0.0172	0.0250	1.0000 [0.6710, 1.3290]
3A relaxed	1.0312 [0.8071, 1.3175]	**0.0007**	**0.0006**	**0.0015**	**0.0022**	1.0000 [0.6421, 1.3579]
2B	0.9851 [0.8923, 1.0876]	**0.0076**	**0.0073**	**0.0132**	**0.0493**	1.0000 [0.7033, 1.2967]
PSM B RF	0.9843 [0.8566, 1.1310]	0.0113	0.0104	0.0199	0.0779	1.0000 [0.5951, 1.4049]
PSM B GLM	0.9793 [0.8562, 1.1201]	0.0105	0.0102	0.0178	0.0555	1.0000 [0.6067, 1.3933]
3B 1–1	0.9543 [0.7344, 1.2400]	0.0181	0.0186	0.0282	0.0528	1.0000 [0.5918, 1.4082]
3B relaxed	0.9565 [0.7269, 1.2586]	0.0305	0.0342	0.0466	0.0748	1.0000 [0.5954, 1.4046]

**Table 5:** Bias-corrected discrimination metrics using rms bootstrap (marked with *) and manual boot-strapped Brier scores. 95% confidence intervals in [ ]. Oversampling with duplication or tripling of the matched cohorts is labeled with “dup” or “tripled”. Best scores among A or B models in bold.

Model	Dxy*	Harrell C*	Uno C*	Brier Score

1	0.2231	0.6115 [0.5907, 0.6336]	0.5813 [0.5591, 0.5980]	0.2295 [0.2222, 0.2381]
2A	0.2402	0.6201 [0.5878, 0.6564]	0.5892 [0.5586, 0.6246]	0.2265 [0.2101, 0.2433]
PSM A RF	0.2402	0.6201 [0.5878, 0.6564]	0.5939 [0.5641, 0.6163]	0.2272 [0.2108, 0.2465]
PSM A GLM	0.2291	0.6145 [0.5746, 0.6455]	0.5883 [0.5502, 0.6229]	0.2285 [0.2131, 0.2488]
3A 1–1	0.3070	0.6535 [0.6073, 0.7150]	0.6422 [0.5846, 0.6971]	0.2070 [0.1764, 0.2530]
3A relaxed	0.3060	0.6530 [0.5865, 0.7275]	0.6247 [0.5685, 0.6777]	0.2178 [0.1719, 0.2723]
3A 1–1 dup	0.3163	0.6582 [0.6132, 0.6996]	0.6642 [0.6251, 0.6960]	0.1983 [0.1765, 0.2252]
3A relaxed dup	0.3196	0.6598 [0.6037, 0.7097]	0.6478 [0.6033, 0.6867]	0.2036 [0.1791, 0.2310]
3A 1–1 triple	**0.3261**	**0.6631** [**0.6309, 0.6952**]	**0.6670** [**0.6344, 0.7017**]	**0.1936** [**0.1736, 0.2156**]
3A relaxed triple	0.3190	0.6595 [0.6178, 0.7004]	0.6538 [0.6223, 0.6831]	0.1986 [0.1780, 0.2231]
2B	0.1534	0.5767 [0.5508, 0.6043]	0.5424 [0.5108, 0.5709]	0.2337 [0.2259, 0.2436]
PSM B RF	0.1594	0.5797 [0.5308, 0.6247]	0.5603 [0.5245, 0.5991]	0.2374 [0.2240, 0.2545]
PSM B GLM	0.1586	0.5793 [0.5310, 0.6230]	0.5401 [0.5067, 0.5759]	0.2480 [0.2354, 0.2646]
3B 1–1	0.2783	0.6392 [0.5626, 0.7181]	0.5857 [0.5257, 0.6474]	0.2328 [0.2040, 0.2651]
3B relaxed	0.3494	0.6747 [0.6057, 0.7460]	0.6247 [0.5534, 0.6879]	0.2386 [0.1917, 0.2891]
3B 1–1 dup	0.2811	0.6406 [0.5829, 0.6957]	0.6126 [0.5645, 0.6628]	0.2228 [0.2042, 0.2549]
3B relaxed dup	0.3786	0.6893 [0.6319, 0.7445]	0.6573 [0.6064, 0.7115]	0.2170 [0.1859, 0.2467]
3B 1–1 triple	0.2952	0.6476 [0.5997, 0.6857]	0.6176 [0.5744, 0.6558]	0.2162 [0.1978, 0.2378]
3B relaxed triple	**0.3802**	**0.6901** [**0.6480, 0.7294**]	**0.6692** [**0.6269, 0.7189**]	**0.2148** [**0.1920, 0.2370**]

**Table 6:** Bias-corrected calibration metrics using rms bootstrap (marked with *) and otherwise manual bootstrap validation with 5-year OS as endpoint. 95% confidence intervals in [ ]. Oversampling with duplication or tripling of the matched cohorts is labeled with “dup” or “tripled”. Best scores among A or B models in bold.

Model	OE Ratio	ICI	E50	E90	Emax	Calibration Slope*

1	0.9404	0.0340	0.0319	0.0643	0.0779	**0.9226** [0.8810, 0.9492]
2A	1.0812	0.0545	0.0635	0.0709	0.0893	0.8453 [0.7628, 0.9069]
PSM A RF	1.0812	0.0545	0.0635	0.0709	0.0893	0.8453 [0.7628, 0.9069]
PSM A GLM	1.0035	0.0321	0.0319	0.0543	0.0618	0.8309 [0.7134, 0.9032]
3A 1–1	0.9760	0.0225	0.0168	0.0416	0.0433	0.6997 [0.5371, 0.8091]
3A relaxed	0.9889	0.0305	0.0287	0.0550	0.0450	0.6326 [0.4665, 0.7733]
3A 1–1 dup	1.0481	**0.0062**	**0.0061**	**0.0068**	**0.0041**	0.8322 [0.7189, 0.8982]
3A relaxed dup	1.0556	0.0251	0.0290	0.0369	0.0370	0.7792 [0.6121, 0.8728]
3A 1–1 tripled	1.0387	−0.0030	−0.0041	−0.0113	−0.0197	0.8784 [0.7359, 0.9407]
3A relaxed tripled	**1.0028**	0.0158	0.0109	0.0409	0.0577	0.8505 [0.6740, 0.9247]
2B	1.0460	0.0210	0.0204	0.0397	0.0484	0.8382 [0.7488, 0.8984]
PSM B RF	0.8693	0.0646	0.0529	0.1076	0.2121	0.7265 [0.5100, 0.8477]
PSM B GLM	0.9799	0.0422	0.0371	0.0831	0.1425	0.7122 [0.4796, 0.8621]
3B 1–1	1.0357	0.0531	0.0579	0.0883	0.1104	0.6215 [0.4157, 0.7910]
3B relaxed	0.8962	0.0755	0.0940	0.1045	0.1064	0.6057 [0.4482, 0.7579]
3B 1–1 dup	1.1285	0.0303	0.0301	0.0566	−0.0206	0.7768 [0.6454, 0.8634]
3B relaxed dup	**1.0043**	0.0413	0.0266	0.1128	0.1356	0.7956 [0.6900, 0.8651]
3B 1–1 tripled	1.0482	**0.0133**	**0.0082**	**0.0171**	**0.0007**	0.8629 [0.7812, 0.9115]
3B relaxed tripled	0.9784	0.0472	0.0388	0.1065	0.1423	0.8645 [0.8064, 0.9076]

## Footnotes

1 Note that the choice of specific values such as the number of strata and cut-off for α will be explained in 3.2.

2
https://www.gurobi.com/

3
https://CRAN.R-project.org/package=rms

4 Harrell’s C index can be calculated from the output vale $D_{xy}$ as $C=\frac{Dxy}{2}+\frac{1}{2}$.
