# Supplementary material for: Improving survival models in healthcare: a novel matching approach
Source: Res Sq. 2024 Dec 12:rs.3.rs-5467577. Preprint. [Version 1] doi: 10.21203/rs.3.rs-5467577/v1 (PMC11661418; doi:10.21203/rs.3.rs-5467577/v1)
Supplement: Supplement 1 [file NIHPPRS5467577V1-supplement-1.pdf]

Appendix

Characteristic	Treated (n (%))	Untreated (n (%))	P-value
**Total Patients**	1197	602	
**Age in years (IQR)**	60 (52.0-67.0)	65.0 (58.0-73.0)	< 0.001
**Sex**			
Male	713 (59.6%)	371 (61.6%)	
Female	484 (40.4%)	231 (38.4%)	0.39899
**Median CEA in µg/L (IQR)**	6.55 (3.0-23.0)	8.8 (3.5-34.5)	0.01496
**Median Diameter of Largest CRLM in cm (IQR)**	3.0 (2.0-4.3)	2.8 (1.9-4.0)	0.50225
**Median Number of CRLMs (IQR)**	2.0 (1.0-3.0)	2.0 (1.0-3.0)	0.65409
**Primary Tumor Grade**			
0	65 (5.4%)	87 (14.5%)	
1	229 (19.1%)	222 (36.9%)	
2	103 (8.6%)	39 (6.5%)	
3	52 (4.3%)	56 (9.3%)	
Unknown	2 (0.2%)	8 (1.3%)	
Missing	746 (62.3%)	190 (31.6%)	< 0.001
**Disease-Free Interval (months)**			
< 12	750 (62.7%)	393 (65.3%)	
≥ 12	447 (37.3%)	207 (34.4%)	0.2374
Missing	0 (0.0%)	2 (0.3%)	
**T Category of Primary Tumor**			
0	1 (0.1%)	4 (0.7%)	
1	53 (4.4%)	8 (1.3%)	
2	203 (17.0%)	58 (9.6%)	
3	661 (55.2%)	372 (61.8%)	
4	268 (22.4%)	145 (24.1%)	
Missing	11 (0.9%)	15 (2.5%)	< 0.001
**Primary Lymph Node Involvement**			
No Metastases	495 (41.4%)	210 (34.9%)	
Metastases	694 (58.0%)	381 (63.3%)	
Missing	8 (0.7%)	11 (1.8%)	0.0132
**Primary Tumor Side**			
Right	559 (46.7%)	169 (28.1%)	
Left	331 (27.7%)	238 (39.5%)	
Rectal	303 (25.3%)	191 (31.7%)	
Missing	4 (0.3%)	4 (0.7%)	< 0.001
**Extrahepatic Disease**			
0.0	1078 (90.1%)	516 (85.7%)	
1.0	119 (9.9%)	86 (14.3%)	
Missing	0 (0.0%)	0 (0.0%)	0.0062
**Surgical Margin Status**			
R0	1050 (87.7%)	445 (73.9%)	
R1	129 (10.8%)	144 (23.9%)	
Missing	18 (1.5%)	13 (2.2%)	< 0.001
**KRAS Mutation**			
0.0	676 (56.5%)	396 (65.8%)	
1.0	521 (43.5%)	204 (33.9%)	
Missing	0 (0.0%)	2 (0.3%)	< 0.001

Table S1: Baseline characteristics of patients with CRLM, treated or untreated with adjuvant chemotherapy before imputation. χ^2-test for categorical variables and Mann-Whitney U test for continuous variables.

**Model**	**Training size**
Model 1	1799
Model 2A	602
Model 2B	1197
Model 3A 1-1	156
Model 3B 1-1	156
Model 3A relaxed	124
Model 3B relaxed	135

Table S2: Training cohort sizes of our different models
